# The Role of Students’ Spiritual Intelligence in Enhancing Their Academic Engagement: A Theoretical Review

**DOI:** 10.3389/fpsyg.2022.857842

**Published:** 2022-05-06

**Authors:** Qiangqiang Ma, Fujun Wang

**Affiliations:** School of Marxism, Jilin University of Finance and Economics, Changchun, China

**Keywords:** academic engagement, positive psychology, spiritual life, student, spiritual intelligence

## Abstract

Spiritual intelligence as a new type of intelligence has been limitedly explored in education. As it connects one’s mental and spiritual life to his/her performance and functioning, it can play an especial role in students’ L2 education. However, few studies have empirically examined this construct in relation to student-related factors like academic engagement. Against this shortcoming, the present mini-review study presented both theoretical and empirical underpinnings of this line of research by defining the concepts, their components, and previous studies. Finally, the study enumerated the existing gaps and offered future directions and implications for the educational practitioners and researchers whose awareness of spiritual intelligence and its impact on L2 education and learner-psychology variables can improve.

## Introduction

Learning a second/foreign language is widely considered as a complicated process involving numerous factors and layers to effectively occur (Benevene et al., 2020). It requires an integration of variables and issues related to both teachers and students. One of the most important drives of L2 learning is taking students’ emotions and diversities into account. This entails an education based on learners’ unique cognitive, affective, and social abilities (Arnold and Fonseca, 2004). Now, it is a common belief among L2 practitioners that language learners in various contexts extensively vary in their personal and instructional profiles and preferences. This proposition came into vogue with a groundbreaking study of multiple intelligences (MIs) by Gardner (1983) who proposed different intelligences for learners to which teachers must pay attention. However, in L2 research, the cognitive aspect of intelligence (IQ) and emotional intelligence (EQ) have dominated the field for decades, especially their impacts and correlations with other L2 learning variables (Elhambakhsh et al., 2018).

Drawing on MI theory, Zohar and Marshall (2001) took a giant step and proposed a new intelligence known as spiritual intelligence (SI, hereafter) that works independently and requires a different understanding of the connection between one’s inner life, mind, and spirit to the external world (Vaughan, 2002). It is an intelligence that focuses on macro-level problem-solving potentials of learners/teachers, particularly problems pertaining to meaning and value of different ways of life (Zohar and Marshall, 2001). It goes beyond a person’s mental ability and conventional psychological development, linking the personal to the transpersonal and the self to spirit (Vaughan, 2002; Estaji and Pourmostafa, 2020). As stated by Denny et al. (2008), SI can establish a learning atmosphere in which students can reach their full potentials as it capitalizes on their ability to make personal meanings out of life experience, consciousness, and critical thinking. In support of these benefits, Zohar (2010) called for an education oriented to the principles of SI that considers students’ curiosity and motivates them to interrogate their previous assumptions about values and events and to embrace new experiences.

Having positioned itself in the body of knowledge in L2 education, SI has recently caught the attention of researchers who mainly explored its impacts and associations with teacher-related variables such as pedagogical success, leadership style, self-regulation, job satisfaction, burnout, professional commitment, and critical thinking ability (Kaur, 2013; Azizi and Azizi, 2015; Zhaleh and Ghonsooly, 2017; Elhambakhsh et al., 2018; Emma et al., 2018; Estaji and Pourmostafa, 2020). With regard to EFL students, there are only a couple of studies that are limited to the role of SI in developing their writing skills and learning strategies (Santoso, 2016; Sotoudehnama et al., 2018) and the impact of SI on EFL students’ classroom behaviors and practices has largely been ignored.

One such overlooked area is the effect of SI on EFL students’ academic engagement that refers to their involvement in classroom activities as a sign of motivation that produces academic energy, zest, investment, and success (Skinner and Pitzer, 2012; Phillips, 2015). As language learning is a complex process, students need to be highly engaged in the classroom so that they can pass the challenges involved in L2 education. This is obtainable by developing their SI level that, in turn, causes more confidence, agency, context-sensitivity, academic achievement (Hassan, 2009). However, the existing literature in this domain lacks empirical/theoretical studies on the correlation between students’ SI and engagement. To fill this gap, the present mini-review study aimed to present the theoretical underpinnings of this line of research and offers future directions concerning EFL students’ SI and academic engagement.

## Background

### Intelligence(s) and Education

**Figure 1:**
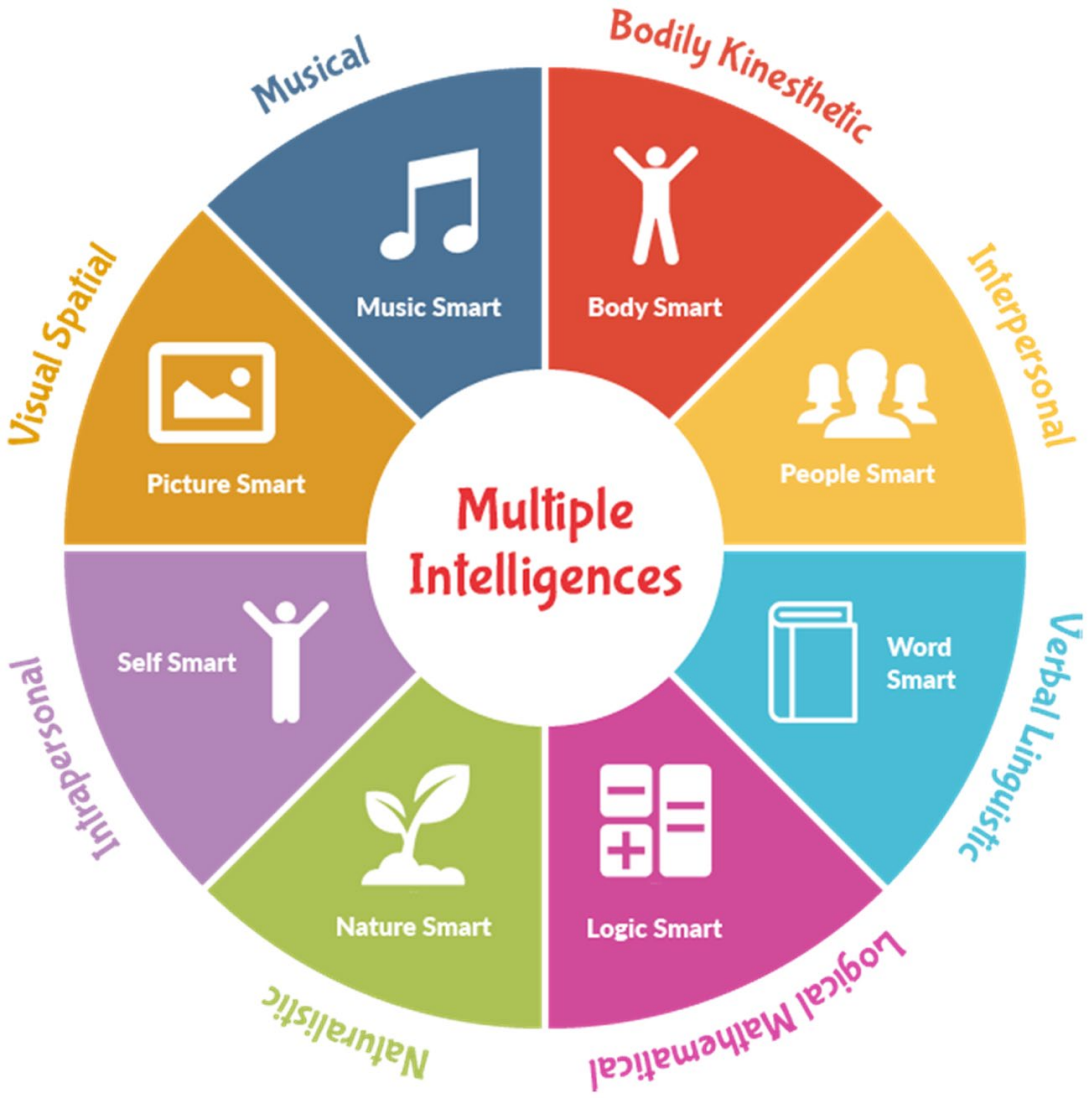
Types of intelligence (Reproduced with permission from Gardner, 1983).

The long history of intelligence in education has witnessed many twists and turns since 1980s when it was first regarded as a fixed and innate feature focusing largely on IQ (Sotoudehnama et al., 2018). Such a simplistic view addressed only one dimension of cognitive ability and ignored others (Hajhashemi et al., 2012). However, with the emergence of Gardner’s (1983) MI model the door was opened for a learner-specific education that cares for learner diversity. For Gardner (1983), intelligence was a combination of several abilities that satisfy individuals’ unique needs and styles. He proposed eight types of intelligence including linguistic, logical/mathematical, spatial, bodily-kinesthetic, musical, interpersonal, intrapersonal, and naturalist (Figure 1).

According to him, musical intelligence concerns one’s sensitivity to the sounds, rhythms, and tones of music, while visual–spatial intelligence pertains to one’s judgment and the ability to visualize through his/her mind’s eye. People with high verbal–linguistic intelligence are skillful in dealing with words and languages, while those with logical-mathematical intelligence are strong in coping with reasoning, numbers, logic, abstractions, and critical thinking. Moreover, he defined bodily-kinesthetic intelligence as being generally good at physical activities like sports, dance, and creating things. Interpersonal intelligence concerns one’s sensitivity to others’ feelings, emotions, intentions, and the ability to work in a group, while interpersonal intelligence means having a deep understanding of the self. Finally, individuals with high naturalistic intelligence have comprehensive knowledge for recognizing and classifying various typologies of plants and animals in the natural world. Despite its universal popularity, Gardner’s (1983) model lacks sufficient empirical data to support the validity of the proposed typologies of intelligence. Additionally, the measurement of these intelligences is yet to be psychometrically approved in education. However, Gardner’s attempts around the theory of MI and existential intelligences paved the way for the introduction of other types of intelligence like emotional intelligence (Goleman, 1995) and SI for the first time in 1990s. Yet, Gardner did not regard SI as a major type of intelligence as it could not meet his eight criteria. Later, at the beginning of the 20th century, the viability of the concept was approved in different disciplines (psychology, general education, medicine), but it is still new to second/foreign language teaching and learning.

### The Concept of Spiritual Intelligence

The construct of SI as a new term in educational psychology refers to the adjustable use of spiritual information to expedite one’s daily problem-solving and goal achievement (Emmons, 2000). It is the capacity to employ and represent spiritual resources to boost everyday performance and wellbeing (Amram and Drye, 2007). It is worth noting that SI is derived from the notion of spirituality and differs from religiosity (Koenig et al., 2000). In education, SI unifies flexibility and emotional resilience and plays a critical role in helping students/teachers make sense of their world and construct aims and values (Zohar and Marshall, 2001). To put simply, SI pertains to one’s capability to behave and perform judiciously and empathetically, while maintaining inner and outer harmony, regardless of the surroundings (Wigglesworth, 2006). To use Gardner’s (2006, p. 20) interpretation, SI is “the intelligence of big questions” that draws on human predisposition to probe fundamental questions about existence. According to Zohar (2010), this type of intelligence can promote one’s motivations for exploration, creativity, cooperation, self-mastery, situational-mastery, and service-provision. In the educational arena, SI concerns the dynamic interplay of students’ or teachers’ inner life of mind, spirit, and their association to instructional experiences and events (Vaughan, 2002). Operationally, SI refers to the use and application of different spiritual information gathered from various resources by EFL/ESL students to improve their academic behavior and classroom practice.

### The Principles and Components Underlying Spiritual Intelligence

In the available literature, many principles and components have been proposed for the construct of SI as a complicated variable related to learners. As a case in point, Zohar (2000) proposed 12 key principles underlying this crucial construct as what follows:

1. Self-awareness: Knowing and recognizing what we believe in, value, and what really motivates us.
2. Spontaneity: Living in the moment.
3. Being vision- and value-led: Acting based on principles and beliefs and living in tune with inspirations.
4. Holism: Seeing things as an integrated system with connected and interwoven parts.
5. Compassion: Having deep empathy.
6. Celebration of diversity: Valuing and accepting others with their differences to form a passionate dialogue.
7. Field independence: Standing against the crowd and having one’s own convictions.
8. Humility: Accepting when we have been wrong and deeply questioning ourselves.
9. Tendency to ask fundamental “why?” questions: This is rooted in our desire to understand and discover things profoundly.
10. Ability to reframe: The ability to see the bigger picture in a wider context to get connected to a larger vision of something.
11. Positive use of adversity: Having courage to admit and learn from mistakes, obstacles, and challenges.
12. Sense of vocation: Doing work with a goal to benefit humanity.

In a similar manner, Emmons (2000) proposed five components for the construct of SI including; (1) the capability to transcend the physical and material; (2) the capability to experience enhanced states of consciousness; (3) the capacity to sanctify everyday experience; (4) the capability to use spiritual resources to solve problems, and (5) the ability to be virtuous (to express gratitude, compassion, forgiveness). Drawing on these, King (2008) added four elements of *critical existential thinking, personal meaning-production, transcendental awareness*, and *conscious-state expansion* to the construct of SI (Table 1).

**Table 1:** Different components of SI.

Component	Description
Critical existential thinking	The ability to critically think of the nature of existence, reality, the universe, space, time, death, and other existential or metaphysical issues
Personal meaning-production	The capability to construct personal meaning and purpose in all physical and mental experiences
Transcendental awareness	The ability to recognize transcendent dimensions of the self, others, and the physical world during the normal, waking state of consciousness, supplemented by the ability to detect their relationship to one’s self and to the physical setting
Conscious-state expansion	The capability to enter and exit higher/spiritual states of consciousness at one’s own discretion

These dimensions indicate that SI is a complex variable that may differ across cultures and contexts. Hence, it can be argued that further empirical studies on this construct in L2 education may come across more and various components. The proposed dimensions are by no means fixed and universal as they have been offered decades ago. Consequently, future empirical studies are suggested to test the existing dimensions or even add/modify them in L2 education, especially in relation to learner psychology variables (e.g., academic engagement, motivation, resilience, efficacy, etc.).

### Student Engagement

As one of the most important objectives of education and what teachers are urgently seeking for, student engagement in language education became more visible and highlighted by positive psychology that gave weight to learners’ positive emotions and inner states (MacIntyre et al., 2019). It is a multi-faceted variable concerning the extent and quality of students’ involvement and participation in classroom tasks and activities (Skinner and Pitzer, 2012; Tu, 2021). Students’ engagement is a representation of their intrinsic motivation that is shaped over time and in a positive academic context (Elliott and Tudge, 2012; Wang and Guan, 2020).

As for the dimensions of this meta-construct, research shows that it encompasses behavioral, emotional, cognitive, agentic, academic, and social dimensions as described in Table 2 (DeVito, 2016; Oga-Baldwin, 2019).

**Table 2:** The dimensions of student engagement.

Dimension	Description
Behavioral	Learners’ compliance and active participation in classroom activities and practices such paying attention, participating in the class, involving in tasks, and doing assignment
Emotional/affective	Learners’ emotional states and affective responses (e.g., fun, anxiety, stress, interest, joy, hope, etc.) to learning events/practices
Cognitive	Learners’ psychological investment in learning and using intricate learning strategies to accomplish a task
Agentic	Learners’ active contribution to the enhancement of learning and teaching processes
Academic	Learners’ psychological and behavioral efforts to acquire academic knowledge and skills
Social	Learners’ engrossment in a range of classroom tasks/activities that intend to stimulate their social interaction and problem-solving abilities

According to Collins (2014), this dynamic and multi-layered variables can be affected by different factors including *phenomenological* factors that are related to one’s ability, culture, and task difficulty; *individual*-*demographic* factors such as age, gender, educational background/qualification; and lastly instructional factors that concern teachers’ classroom practices and behaviors. However, SI as an individual-demographic factor that highlights the diversity of learners and the role of spirituality and mind in learning has not been studied sufficiently as a factor influencing academic engagement.

### Previous Studies

Contrary to other areas of research on learner-psychology and intelligence, scant studies exist considering students’ SI and its role in L2 education. This might be due to the unclear conceptualization of the term and its association to SLA. Likewise, the concept seems to be more fitting fields that are religion-oriented (Emmons, 2000; Koenig et al., 2000). Nevertheless, some scholars have broken the ice and begun exploring the construct of SI in SLA over the past years arguing that it can promote teachers’ pedagogical success, self-regulation, job satisfaction, burnout, professional commitment, and critical thinking ability (Kaur, 2013; Azizi and Azizi, 2015; Zhaleh and Ghonsooly, 2017; Elhambakhsh et al., 2018; Emma et al., 2018). Additionally, in their recent mixed-methods study, Estaji and Pourmostafa (2020) examined the relationship between Iranian EFL teachers’ SI and leadership style in light of their teaching experience level. In so doing, 236 EFL teachers completed two questionnaires on these two variables out of which 10 teachers were later interviewed. The results indicated that the components of SI significantly predicted novice and experienced EFL teachers’ perceptions of leadership styles. Likewise, Emma et al. (2018) quantitatively investigated the effect of SI on Indonesian teachers’ teamwork and commitment using a questionnaire that was completed by 143 teachers. The results of correlation analysis revealed a direct impact of SI on participants’ teamwork and commitment.

As for the effect of SI on students’ classroom behaviors and practices like their degree of engagement in/with classroom activities, Smartt (2014) argued that SI and its features have a strong influence on American secondary students’ engagement and performance. Moreover, the role of SI in shaping EFL students’ language learning has caught the attention of some studies that identified that SI improves EFL students’ writing skills and learning strategies (Santoso, 2016; Sotoudehnama et al., 2018). Furthermore, research displays that students’ engagement can cause diverse positive academic outcomes (Eccles, 2016) and is correlated with achievement, motivation, interpersonal skills, psychosocial adjustment, psychological safety, effective learning, success, and classroom culture (Chase et al., 2015; Jang et al., 2016; Wang, 2017; Tu, 2021; Xie and Derakhshan, 2021). Despite these studies, empirical research on the association of student engagement and SI, as two constructs in learner-psychology, is demanded in various EFL/ESL contexts. Moreover, this area lacks sufficient research on the intersection of intelligence and positive emotions which have tight connections on the part of learners and their learning. Hence, running explorations in this strand of research is highly recommended.

## Concluding Remarks

In this mini-review study, it was maintained that SI can play a pivotal role in shaping and raising EFL students’ academic engagement in the class. Hence, it can offer implications for different stakeholders as their knowledge of intelligence and positive emotions adds fresh insights to their practices. This theoretical review is meritorious to EFL teachers in that they can work on techniques by which they can improve their pupils’ SI and engagement levels. They can also get familiar with the role of learner-related variables (SI) and emotions in the process of learning. Similarly, EFL students can benefit from this study in that it can increase their awareness of the power of their mental/spiritual resources to solve problems and experience well-being in education. Teacher trainers, as another group, may find this study valuable and offer training courses to EFL teachers in which ways of dealing with and improving students’ SI and engagement are fully explained and practiced. Additionally, they can add a psycho-emotional aspect to their training programs along with pedagogical issues. Furthermore, this article can be useful for L2 researchers and inspire them to run similar and complementary studies in which the role of SI in SLA is more illustrated. The current state of research in this domain is limited to a number of correlational studies using a one-shot design. Hence, future studies can be conducted using qualitative and mixed-methods designs. Moreover, most of the studies on SI have focused on teacher-related variables, so future research can be recommended examining learner-related variables especially those of PP. As both SI and engagement are dynamic and multi-faceted, experimental studies are also suggested to see if they develop over a course or not. Finally, cross-cultural examinations can be done of EFL students’ level of SI and engagement to identify whether cultural and social factors mediate their relationship.

## Author Contributions

All authors listed have made a substantial, direct, and intellectual contribution to the work, and approved it for publication.

## Conflict of Interest

The authors declare that the research was conducted in the absence of any commercial or financial relationships that could be construed as a potential conflict of interest.

## Publisher’s Note

All claims expressed in this article are solely those of the authors and do not necessarily represent those of their affiliated organizations, or those of the publisher, the editors and the reviewers. Any product that may be evaluated in this article, or claim that may be made by its manufacturer, is not guaranteed or endorsed by the publisher.
